# Thrombotic genetic risk factors and warfarin pharmacogenetic variants in São Miguel's healthy population (Azores)

**DOI:** 10.1186/1477-9560-7-9

**Published:** 2009-06-18

**Authors:** Claudia C Branco, Tânia Pereirinha, Rita Cabral, Paula R Pacheco, Luisa Mota-Vieira

**Affiliations:** 1 Molecular Genetics and Pathology Unit, Hospital of Divino Espirito Santo of Ponta Delgada, EPE, São Miguel Island, Azores, Portugal; 2 Instituto Gulbenkian de Ciência, Oeiras, Portugal

## Abstract

**Background:**

The Azorean population presents the highest standardized mortality rate for cardiovascular diseases (CVD) when compared to mainland Portugal and other populations. Since thrombosis is a common cause of CVD, we assessed four polymorphisms in three thrombotic risk genes – *F5 *(G1691A), *F2 *(G20210A) and *MTHFR *(C677T, A1298C), in 469 healthy blood donors from São Miguel Island (Azores). We also analysed the *CYP2C9 *(C430T, A1075C) and *VKORC1 *(G1639A) variants in fifty-eight individuals with predisposition to thrombosis (possessing at least one variation in *F5 *or *F2 *genes and one in *MTHFR*) to evaluate their warfarin drug response genetic profiles.

**Results:**

Among the 469 individuals, the data showed that thrombotic risk allele frequencies – 1691A (4.9%), 20210A (1.8%), 677T (41.7%) and 1298C (24.8%) – were similar to other Caucasians, but significantly different from mainland Portuguese (χ^2^, *p *< 0.001). The combined analysis of these variants identified twenty-two different genetic profiles (genotype order: *F5*, *F2*, *MTHFR *C677T and A1298C). Complete homozygosity for all wild-type alleles (GG GG CC AA) was present in 11.7%, being GG GG CT AA (22.4%) the most frequent profile. The results also demonstrated that 12.4% (58 out of 469) of São Miguel islanders have increased genetic predisposition to thrombosis. Subsequently, we evaluated these individuals for their warfarin response genetic profiles. The data showed that seven out of fifty-eight individuals are poor metabolizers (two with CYP2C9*2/*2 and five with CYP2C9*2/*3 genotypes). *VKORC1 *polymorphism analysis identified twelve individuals (20.7%) with AA genotype, who probably will require lower doses of warfarin. The joint analysis of *CYP2C9 *and *VKORC1 *revealed that 79.3% (46 out of 58) of the individuals carry at least one polymorphism in these genes. Within these, twenty-five individuals (43.1%) need intermediate and/or low doses of warfarin, if treatment is started.

**Conclusion:**

The present study demonstrated, for the first time, that São Miguel, and possibly the Azores population, shows significant differences on allele frequencies of thrombotic risk factors when compared to mainland Portugal. This research constitutes a primary approach for future studies on CVD, as well as for the implementation of warfarin dosing protocols using the patient's genotypic information.

## Background

Cardiovascular diseases (CVD) are complex disorders that include dysfunctional conditions of the heart, arteries and veins. Within these diseases, thrombosis is the third most common cardiovascular event worldwide [1]. It can derive from: i) genetic factors; ii) acquired changes in the clotting mechanism; and iii) more commonly, interaction between the two factors described above [2-4]. The main hereditary form of thrombosis is caused by the G1691A variation in the factor V Leiden gene (*F5*; 1q23), resulting in resistance to activated protein C (APC). In the coagulation biochemical pathway, APC prevents blood clots from growing extensively large, by inactivating coagulation factor V (encoded by *F5*). The G1691A variation may increase the risk for thrombosis by 8 and 91 in heterozygous and homozygous states, respectively [5]. According to Poort *et al. *[6], prothrombin (*F2*; 11p11-q12) represents the second most important inherited thrombotic risk factor. A single base substitution (G20210A) in the *F2 *3'-untranslated region causes elevated plasma prothrombin levels. The same authors reported a 2.8-fold increase risk for venous thrombosis in heterozygous and homozygous variant individuals [6]. Other reported gene involved in thrombosis, the methylenetetrahydrofolate reductase (*MTHFR*; 1p36.3), includes two variants – C677T and A1298C [7]. The 677T alters the protein's folate binding site and lowers the enzymatic activity of MTHFR. As consequence, patients have elevated plasma levels of total homocysteine (tHcy) [8]. The 1298C variant also decreases the enzymatic activity of MTHFR, which is more pronounced in homozygous variant than in heterozygous state [8]. According to Heijer *et al. *[9], individuals with the 677TT genotype show a 15% (OR = 1.1; 95% CI = 1.0–1.3) increased risk for venous thromboembolism (VTE) in Europe. Additionally, Klerk *et al. *[10] reported an OR of 1.3 (95% CI = 1.1–1.5) for the 677CT genotype.

The genetic characterization of the individual's potential risk for developing thrombosis and response to drugs is essential in clinical follow-ups, as well as for medical prescription. Warfarin is one of the most widely used anticoagulant drug, which requires a thorough risk-benefit analysis since the dose prescribed should avoid hemorrhagic complications and achieves suppression of thrombosis [11]. The administrated drug is a racemic mixture of S- and R-enantiomers, having S- the majority of the therapeutic effect [12]. Warfarin pharmacogenetic studies demonstrated that variants in the *CYP2C9 *(Cytochrome P450 2C9) and *VKORC1 *(Vitamin K epoxide reductase complex subunit 1) genes account for approximately 50–60% of drug dosing variability [13,14]. Cytochrome P450 2C9 is the major enzyme responsible for metabolising the active S-enantiomer [12]. Although there are many polymorphisms in *CYP2C9*, the most frequent variants are: CYP2C9*1 (Arg144/Ile359, wild-type), CYP2C9*2 (Arg144Cys) and CYP2C9*3 (Ile359Leu). These last two are associated with decreased metabolic efficiency of the CYP2C9 enzyme and increased risk of bleeding when administrated initial dosages of warfarin [15]. A G>A variation at position 1639 in the promoter region of the *VKORC1 *gene results in decreased mRNA transcription and increased sensitivity to warfarin inhibition of hepatic synthesis of functional vitamin K-dependent coagulation factors [13]. Rieder *et al. *[13] studied *VKORC1 *polymorphisms and classified individuals according to warfarin dose requirements into distinct groups: high (GG), intermediate (GA) and low (AA).

The Azores population presents the highest standardized mortality rate caused by CVD when compared to mainland Portugal (Portuguese General Directorate of Health) [16] and other populations. Since thrombosis is a known cause of CVD, the present research aims to assess, in the São Miguel healthy population, the allelic variants and genotypes of three genes – *F5*, *F2 *and *MTHFR *– involved in blood clot formation. Moreover, in the individuals with predisposition to thrombosis, we evaluated the pharmacogenetic profile for warfarin, by characterizing *CYP2C9 *and *VKORC1 *polymorphisms. This information is important for the implementation of warfarin pre-prescription genotyping with the goal of performing individualized treatment.

## Materials and methods

### Population sample

The population sample comprised a group of 469 São Miguel Island blood donors from the anonymized Azorean DNA bank located in the main Hospital of Azores archipelago, Portugal [17]. São Miguel, the largest island in this archipelago, has 131,609 inhabitants (54.4% of the Azoreans; 2001 Census, Portugal National Institute of Statistics) [18]. In this group we analysed four polymorphisms in three thrombotic predisposition genes – *F5 *(G1691A), *F2 *(G20210A) and *MTHFR *(C677T, A1298C). After evaluating the genetic variants in the aforementioned genes, we selected a subset of individuals who met one of the following criteria: 1. one variant in *F5 *and one in *MTHFR *(fourty-three individuals); 2. one variant in *F2 *and one in *MTHFR *(fifteen individuals) and 3. one variant in *F5*, *F2 *and *MTHFR *(zero individuals). These criteria were chosen to restrict the sample to individuals who would have a higher genetic risk for a thrombotic event, and hence fifty-eight individuals were analysed for *CYP2C9 *and *VKORC1 *polymorphisms.

### Genotyping of *F5 *and *MTHFR *polymorphisms

*F5 *and *MTHFR *polymorphisms (Table 1) were evaluated by polymerase chain reaction – restriction fragment length polymorphism (PCR-RFLP), using three different restriction enzymes: *Mnl*I, *Hinf*I and *Mbo*II (New England Biolabs, Beverly, MA, USA). The genotyping protocol for the detection of the *F5 *polymorphism and *MTHFR *variants was adapted from Kumar *et al*. [19] and Skibola *et al*. [20], respectively.

**Table 1:** Main characteristics of the seven genetic variants analysed in this study

**Polymorphism**	**Allele variant**	**Amino acid change**	**Gene**		
			
			Location	Position	NCBI dbSNP rs#
Factor V Leiden	1691G>A	R506Q	1q23	Exon 10	rs6025
Prothrombin	20210G>A	NA	11p11-q12	3'-UTR	rs1799963
*MTHFR*	677C>T	A222V	1p36.3	Exon 5	rs1801133
	1298A>C	E429A	1p36.3	Exon 8	rs1801131
CYP2C9*2	430C>T	R144C	10q24.1	Exon 3	rs1799853
CYP2C9*3	1075A>C	I359L	10q24.1	Exon 7	rs1057910
*VKORC1*	1639G>A	NA	16p11.2	Promoter	rs9923231

NA – not applicable; UTR – untranslated region.

### Genotyping of *F2*, *CYP2C9 *and *VKORC1 *polymorphisms

The genetic tests used for *F2*, *CYP2C9 *and *VKORC1 *(Table 1) were PTH StripAssay™ and PGX-Thrombo StripAssay™ kits (ViennaLab-Labordiagnostika GmbH-Austria), according to the manufacturer's recommendations. These assays are based on reverse hybridization system, using biotin-labelled amplification products to a parallel array of allele-specific oligonucleotides immobilized on a membrane test strip.

### Statistical Analysis

Allele frequencies were obtained by direct count. Hardy-Weinberg equilibrium was calculated using the Arlequin software v.3.20. This software was used to assess maximum likelihood genetic profiles for thrombotic risk factors, through the expectation maximization algorithm, an iterative procedure from multilocus genotype data with unknown gamete phase. Allele and genotype frequencies were compared between São Miguel islanders and mainland Portuguese by χ^2 ^test using the SPSS software v.17.0. A *p *< 0.05 was considered statistically significant.

## Results

### Allele and genotype frequencies of thrombotic genetic risk factors

Allele and genotype frequencies of *F5 *(G1691A), *F2 *(G20210A) and *MTHFR *(C677T, A1298C) polymorphisms were assessed in 469 healthy individuals from São Miguel Island, Azores (Table 2). All markers were in Hardy-Weinberg equilibrium. The *F5 *variant 1691A frequency was 4.9%, whereas the heterozygous genotype (1691GA) was present at 9.8% (fourty-six individuals). We did not detect 1691AA individuals. The results on the *F2 *analysis show an allele frequency of 1.8% for the 20210A variant. Although this polymorphism is also uncommon in other populations, one homozygous variant individual (0.2%) and fifteen heterozygous (3.2%) were observed in the present study. The frequency of the variant alleles in *MTHFR *(677T and 1298C) was 41.7 and 24.8%, respectively. Overall, 17.9% are 677TT (eighty-four individuals), and 6.0% present the 1298CC genotype (twenty-eight individuals).

**Table 2:** Allele and genotype frequencies for thrombotic risk factors and for warfarin pharmacogenetic variants in São Miguel population

**Polymorphism**	Allele	n^a^	Freq.	Genotype	n^b^	Freq.
**Thrombotic risk variants in general population (N = 469)**						
***Factor V Leiden***						
1691G>A	G	892	0.951	GG	423	0.902
	A	46	0.049	GA	46	0.098
				AA	0	0.000
						
***Prothrombin***						
20210G>A	G	921	0.982	GG	453	0.966
	A	17	0.018	GA	15	0.032
				AA	1	0.002
						
***MTHFR***						
677C>T	C	547	0.583	CC	162	0.345
	T	391	0.417	CT	223	0.475
				TT	84	0.179
						
1298A>C	A	705	0.752	AA	264	0.563
	C	233	0.248	AC	177	0.377
				CC	28	0.060

**Warfarin pharmacogenetic variants in individuals with predisposition to thrombosis (N = 58)**						
***CYP2C9***						
430C>T^c^	C	98	0.845	*1/*1	35	0.603
	T	18	0.155	*1/*2	9	0.155
				*2/*2	2	0.035
						
1075A>C^c^	A	104	0.897	*1/*3	7	0.121
	C	12	0.103	*2/*3	5	0.086
				*3/*3	0	0.000
						
***VKORC1***						
1639G>A	G	66	0.569	GG	20	0.345
	A	50	0.431	GA	26	0.448
				AA	12	0.207

^a ^Number of chromosomes

^b ^Number of alleles

^c^*1*1 (430CC/1075AA), *1*2 (430CT/1075AA), *1*3 (430CC/1075AC), *2*2 (430TT/1075AA), *2*3 (430CT/1075AC) and *3*3 (430CC/1075CC)

**Figure 1:**
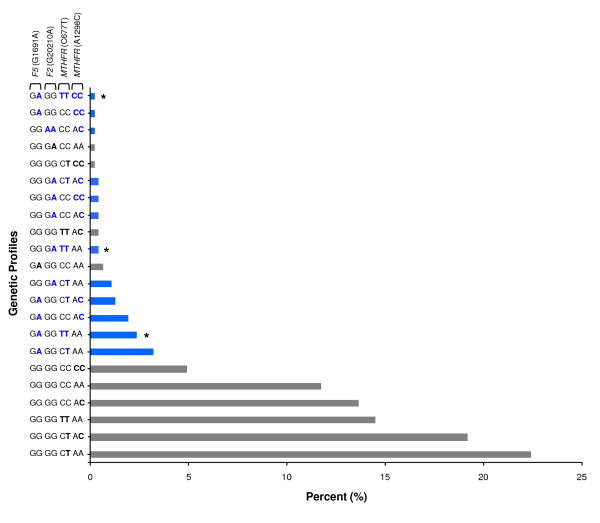
**Combined genotypes for thrombotic risk factors – *F5*, *F2 *and *MTHFR *– in São Miguel population**. Twenty-two different genetic profiles were obtained. Bold characters (blue and black) indicate nucleotide changes compared to the wild-type allele. The asterisk (*) represents the combination of the *F5 *G1691A or *F2 *G20210A with *MTHFR *677TT genotype. Blue bars correspond to the subset of fifty-eight individuals with genetic predisposition to thrombosis selected for warfarin pharmacogenetic study.

Twenty-two different genetic profiles for *F5*, *F2 *and *MTHFR *(order in genotype profile) were observed in São Miguel's population (Figure 1). The frequency of individuals who present a wild-type genotype for all polymorphisms (GG GG CC AA; 11.7%) was almost half of the major profile (GG GG CT AA; 22.4%), differing in heterozygosity for *MTHFR *677CT. Statistical analysis of the combined genotypes demonstrated that there is no significant difference between the present distribution and the hypothetical obtained by Arlequin software (χ^2^, *p *= 0.99). No heterozygous or homozygous profiles for all four variants were observed. Almawi *et al. *[21], reported an OR of 10.5 (95% CI = 4.3–25.3) or 6.3 (95% CI = 1.5–26.0) for joint occurrence of the *F5 *G1691A or *F2 *G20210A with *MTHFR *677TT genotype, respectively, enhancing the risk for deep vein thrombosis (DVT). Based on this criterion, fifteen (3.2%, *F5*/*MTHFR*) and two individuals (0.4%, *F2*/*MTHFR*) have higher risk for DVT development (Figure 1 – asterisk character; Table 3 – bold characters). In general, the joint analysis of all four variants demonstrated that 12.4% of the total sample (58 out of 469) has increased predisposition to thrombosis development.

**Table 3:** Warfarin pharmacogenetic profile obtained in the fifty-eight individuals, carriers of at least one variation in *F5 *or *F2 *genes and one in *MTHFR*

	**Thrombotic risk factors**				**Warfarin pharmacogenetics**		
		
	*F5*	*F2*	*MTHFR*		*CYP2C9*		*VKORC1*
					
**N (%)**	1691G>A	20210G>A	677C>T	1298A>C	430C>T	1075A>C	1639G>A
**1 (1.7)**	**G*A***	**GG**	***TT***	**AA**	***TT***	**A*C***	**G*A***
**1 (1.7)**	**G*A***	**GG**	***TT***	**AA**	**C*T***	**A*C***	***AA***
1 (1.7)	G*A*	GG	CC	A*C*	C*T*	A*C*	G*A*
2 (3.5)	G*A*	GG	C*T*	AA	C*T*	A*C*	G*A*
1 (1.7)	G*A*	GG	CC	A*C*	C*T*	A*C*	GG
1 (1.7)	GG	G*A*	C*T*	A*C*	CC	A*C*	*AA*
1 (1.7)	G*A*	GG	CC	*CC*	CC	A*C*	G*A*
1 (1.7)	G*A*	GG	C*T*	A*C*	CC	A*C*	G*A*
**1 (1.7)**	**GG**	**G*A***	***TT***	**AA**	**CC**	**A*C***	**G*A***
1 (1.7)	G*A*	GG	CC	A*C*	CC	A*C*	GG
2 (3.5)	G*A*	GG	C*T*	AA	CC	A*C*	GG
**1 (1.7)**	**G*A***	**GG**	***TT***	**CC**	***TT***	**AA**	**G*A***
1 (1.7)	G*A*	GG	C*T*	AA	C*T*	AA	*AA*
1 (1.7)	G*A*	GG	C*T*	A*C*	C*T*	AA	G*A*
**1 (1.7)**	**G*A***	**GG**	***TT***	**AA**	**C*T***	**AA**	**G*A***
2 (3.5)	GG	G*A*	C*T*	A*C*	C*T*	AA	G*A*
1 (1.7)	GG	G*A*	C*T*	AA	C*T*	AA	GG
2 (3.5)	G*A*	GG	C*T*	AA	C*T*	AA	GG
**1 (1.7)**	**G*A***	**GG**	***TT***	**AA**	**C*T***	**AA**	**GG**
2 (3.5)	G*A*	GG	CC	A*C*	CC	AA	*AA*
1 (1.7)	G*A*	GG	C*T*	AA	CC	AA	*AA*
**3 (5.2)**	**G*A***	**GG**	***TT***	**AA**	**CC**	**AA**	***AA***
1 (1.7)	GG	G*A*	CC	A*C*	CC	AA	*AA*
1 (1.7)	GG	G*A*	C*T*	A*C*	CC	AA	*AA*
**1 (1.7)**	**GG**	**G*A***	***TT***	**AA**	**CC**	**AA**	***AA***
2 (3.5)	G*A*	GG	CC	A*C*	CC	AA	G*A*
1 (1.7)	G*A*	GG	C*T*	AA	CC	AA	G*A*
2 (3.5)	G*A*	GG	C*T*	A*C*	CC	AA	G*A*
**4 (6.9)**	**G*A***	**GG**	***TT***	**AA**	**CC**	**AA**	**G*A***
1 (1.7)	GG	*AA*	CC	A*C*	CC	AA	G*A*
1 (1.7)	GG	G*A*	CC	A*C*	CC	AA	G*A*
1 (1.7)	GG	G*A*	CC	*CC*	CC	AA	G*A*
2 (3.5)	GG	G*A*	C*T*	AA	CC	AA	G*A*
2 (3.5)	G*A*	GG	CC	A*C*	CC	AA	GG
3 (5.2)	G*A*	GG	C*T*	AA	CC	AA	GG
2 (3.5)	G*A*	GG	C*T*	A*C*	CC	AA	GG
1 (1.7)	GG	G*A*	CC	*CC*	CC	AA	GG
1 (1.7)	GG	G*A*	C*T*	AA	CC	AA	GG
**3 (5.2)**	**G*A***	**GG**	***TT***	**AA**	**CC**	**AA**	**GG**

Italic characters indicate nucleotide changes considering the wild-type allele and in bold illustrate carriers of the *F5 *G1691A or *F2 *G20210A with *MTHFR *677TT genotype conferring high thrombotic risk.

**Figure 2:**
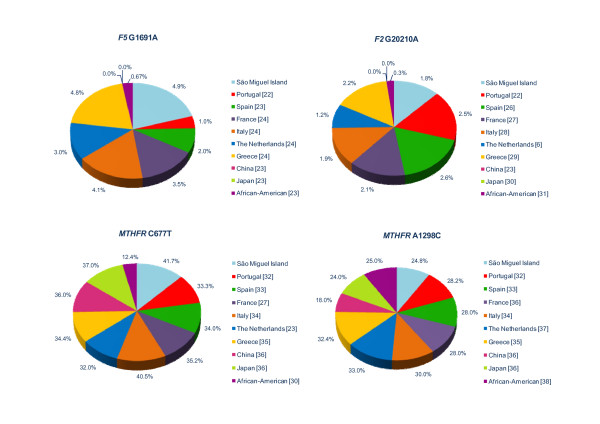
**Allele frequencies of the thrombotic risk factors – *F5 *(G1691A), *F2 *(G20210A) and *MTHFR *(C677T, A1298C) – in different populations, including São Miguel**.

The comparison of allele frequencies of the thrombotic genetic risk factors – *F5*, *F2 *and *MTHFR *– in different populations, including São Miguel, is depicted in Figure 2[6,22-38]. *F5 *G1691A varies from as low as 0.67% (African-American) to 4.9% (São Miguel Island). This trend is not evident for *F2 *G20210A, where the values present a lower range, from 0.3% for African-American to 2.6% for Spanish. African populations showed the lowest frequency for *MTHFR *variants. Taken together, the results demonstrate that, in São Miguel, the frequencies for all polymorphisms were similar to those found in Caucasians.

### Frequencies of pharmacogenetic variants in individuals with genetic predisposition to thrombosis

In order to understand warfarin pharmacogenetic profile in the fifty-eight individuals, carriers of at least one variation in *F5 *or *F2 *genes and one in *MTHFR*, we studied *CYP2C9 *and *VKORC1 *gene polymorphisms. The allele frequencies for CYP2C9*2 and CYP2C9*3 were 15.5 and 10.3%, respectively (Table 2). Five genotypes were observed: CYP2C9*1/*1 (60.3%), CYP2C9*1/*2 (15.5%), CYP2C9*1/*3 (12.1%), CYP2C9*2/*2 (3.5%) and CYP2C9*2/*3 (8.6%). The data indicate that 39.7% will require intermediate and/or low warfarin maintenance doses. Regarding the *VKORC1 *gene (G1639A), we identified twenty (34.5%) wild-type individuals, twenty-six (44.8%) heterozygous and twelve (20.7%) homozygous variant, corresponding to an allele frequency of 43.1% for 1639A (Table 2). These results suggest that 65.5% need intermediate and/or low doses of warfarin.

The majority of individuals with a genetic predisposition to thrombosis (79.3%, 46 out of 58) carry at least one polymorphism in the genes involved in the metabolism of warfarin (Table 3). Moreover, the joint analysis of *CYP2C9 *and *VKORC1 *identified a total of thirty-three (57%) individuals that probably will require high doses of warfarin (genotypes: *1*1-GG→twelve individuals, *1*1-GA→fourteen, *1*2-GG→four and *1*3-GG→three), and eight (13.8%) will need lower doses (genotypes: *1*2-AA→one individual, *1*3-AA→one, *2*2-GA→two, *2*3-GA→one and *2*3-AA→three). The remaining seventeen individuals (29.3%) present one of the following genotypes: *1*1-AA→nine individuals, *1*2-GA→four, *1*3-GA→three and *2*3-GG→one, where all of them will possibly necessitate intermediate doses. In summary, twenty-five individuals (43.1%) will need intermediate and/or low doses of warfarin, if treatment is started.

## Discussion

In the general population, individuals may develop thrombosis due to multiple risk factors, acquired and genetic. Clearly, acquired factors (smoking, high cholesterol, obesity, etc.) are more frequent and combined with a genetic predisposition further enhance the risk of a thrombotic event. In the present study, the results obtained from 469 São Miguel healthy individuals demonstrate that approximately 5% have the *F5 *1691A allele, the most common hereditary risk factor. Although the frequency found for São Miguel is higher than mainland Portuguese, 1% [22], we did not detect homozygous variant individuals. Nonetheless, the data suggest that São Miguel islanders have an increased predisposition to thrombosis and, most probably for CVD. Another inherited cause of thrombosis is the G20210A polymorphism in the *F2 *gene. According to Bosler *et al. *[39], this variant is weak but a consistent risk factor for DVT and for development of CVD, although to a lesser degree than *F5 *[4]. The *F2 *results show a lower frequency, 1.8%, when compared to mainland Portugal, 2.5% [22].

The most general form of genetic hyperhomocysteinemia results from the *MTHFR *C677T variant, which has a relatively high frequency throughout the world and is considered a low risk factor for vascular diseases [9]. In São Miguel population, *MTHFR *677T shows an allelic frequency of 41.7%, very similar to Europeans (Figure 2). According to Almawi *et al. *[21], *MTHFR *677TT genotype when associated with *F5 *or *F2 *increases the risk of DVT [20]. Based on these genotypes, the data suggests that 3.6% (seventeen individuals) of the São Miguel Island population may have higher risk for developing a thrombotic event. Regarding the 1298C variant, the results were in agreement with those reported by other studies (Figure 2), where the polymorphism is also common. We can hypothesise that if the 1298CC individuals have higher plasma homocysteine levels, they will present an enhanced risk for thrombosis.

The joint analysis of all variants demonstrated that 12.4% of the total sample (58 out of 469) has increased predisposition to thrombosis development. In addition, comparison of allele frequencies for all thrombotic risk factors between São Miguel and mainland Portugal revealed statistically significant differences (χ^2^, *p *< 0.001). Family-based investigation tends to have biased relative risk estimation, because oversampling of affected individuals is normally present. Therefore, the genetic characterization of disease variants in the general population is important to correct this potential bias. Consequently, the present research will aid the clinical follow-ups of patients with recurrent thrombotic events.

In today's medicine, the association between pharmacogenetics and drug therapy represents an important tool to treat or control complex diseases. Drug treatment is based on the assumption that a particular dose will yield a predefined blood concentration in circulation and, thereby, establish the desired therapeutic effect [40]. Recent investigations clearly demonstrate an association between warfarin dose requirements and genetic variations in *CYP2C9 *and *VKORC1 *[14]. Here, we analysed 58 individuals with genetic predisposition to thrombosis defined by at least one variation in *F5 *or *F2 *genes and one in *MTHFR*. The results obtained show that approximately 60.3% present the genotype CYP2C9*1/*1, while the remaining have CYP2C9*2 and CYP2C9*3 variants. The presence of two copies of the active CYP2C9*1 alleles represents the normal metabolic capacity – extensive metabolizers (EMs) -, indicating that individuals with this genotype require the highest warfarin maintenance doses [41].

Considering São Miguel islanders with genetic predisposition to thrombosis, the data of CYP2C9*2 and CYP2C9*3 alleles demonstrate a close similarity with different European populations [42-44]. Individuals who are heterozygous (CYP2C9*1/*2 and CYP2C9*1/*3) represent a middle range of metabolic activity – intermediate metabolizers, IMs [43,44]. The results reveal that, within the fifty-eight subset, 15.5 and 12.1% are IMs, respectively. The CYP2C9*2 and CYP2C9*3 variants have less activity and metabolize warfarin more slowly. Individuals with one or two of these variations – poor metabolizers (PMs) – have an enhanced response to warfarin, as well as an increased risk of bleeding when beginning and during treatment if not genotyped previously [44]. We observed that seven out of fifty-eight individuals are PMs (two with CYP2C9*2/*2 and five with CYP2C9*2/*3 genotypes; Table 2). Detecting genetic variations in drug-metabolizing enzymes is useful for identifying individuals who may experience adverse drug reactions (ADRs) with conventional medication doses. Those who are *CYP2C9 *PMs may exhibit different pharmacokinetics than wild-type individuals if they are medicated with warfarin. Consequently, these individuals possibly will require non-conventional doses of drugs that need CYP2C9 enzyme for biotransformation [45].

Polymorphisms within *VKORC1*, a target of warfarin inhibition, may explain 23% of this drug dosage variability [13]. Rieder and colleagues [13] divided individuals into warfarin high-dose for 1639GG genotype, intermediate-dose for 1639GA and low-dose for 1639AA. This variability is attributed to the fact that the variant lies within the *VKORC1 *promoter, potentially resulting in decreased transcription of the gene product. In general, the results suggest that 65.5% of the fifty-eight individuals with predisposition to thrombosis will require intermediate and/or low doses of warfarin, if treatment is started. Personalized medicine relies on data based on the human genome and disease biology characterization for the development of preventive, diagnostic, and therapeutic strategies that target the underlying determinants of disease in populations with specific molecular profiles [46]. For this reason, the knowledge obtained from the pharmacogenetic profile in the São Miguel's general population is and will be important for the development of patient's individualized therapy, mainly for those who have difficulty to stabilize the warfarin dose.

## Conclusion

The identification of hereditary risk factors, with appropriate clinical evaluation will allow the informed patient and physician to work together for effective management of thrombosis and prevention of subsequent thrombotic events. The present study demonstrated, for the first time, that São Miguel, and possibly the Azores population, shows significant differences on allele frequencies of thrombotic genetic risk factors when compared to mainland Portugal. This research constitutes a primary approach for future studies on CVD, as well as for the implementation of warfarin dosing protocols using the patient's genotypic information.

## Abbreviations

ADRs: Adverse drug reactions; APC: Activated protein C; CI: Confidence interval; CVD: Cardiovascular diseases; *CYP2C9*: *C*ytochrome P450 2C9 gene; DVT: Deep vein thrombosis; EMs: Extensive metabolizers; *F2*: Prothrombin gene; *F5*: Factor V Leiden gene; IMs: Intermediate metabolizers; *MTHFR*: Methylenetetrahydrofolate reductase gene; OR: Odds Ratio; PCR-RFLP: Polymerase chain reaction – restriction fragment length polymorphism; PMs: Poor metabolizers; SNP: Single nucleotide polymorphism; tHcy: Total homocysteine; *VKORC1*: Vitamin K epoxide reductase complex subunit 1 gene; VTE: Venous thromboembolism.

## Competing interests

The authors declare that they have no competing interests.

## Authors' contributions

CCB and TP, contributed equally, by performing the experiments, statistical analysis and drafting the manuscript. RC genotyped *MTHFR *variants and PRP participated in the analysis. LMV designed the study, provided scientific orientation and revised the manuscript. All authors read and approved the final manuscript.
